# Addressing mental health needs in low- and middle-income countries: the case of São Caetano do Sul, Brazil

**DOI:** 10.47626/2237-6089-2023-0674

**Published:** 2025-11-05

**Authors:** Flavia Ismael, Artur Ramos, Rafael Erik de Menezes, Regina Maura Zetone Grespam, Cibele Cristine Remondes Sequeira, Jair de Jesus Mari, João Mauricio Castaldelli-Maia

**Affiliations:** 1 Seção de Saúde Mental Secretaria de Saúde São Caetano do Sul SP Brazil Seção de Saúde Mental, Secretaria de Saúde, São Caetano do Sul, SP, Brazil.; 2 Universidade Municipal de São Caetano do Sul São Caetano do Sul SP Brazil Universidade Municipal de São Caetano do Sul (USCS), São Caetano do Sul, SP, Brazil.; 3 Centro de Atenção Psicossocial Álcool e Drogas Butantã São Paulo SP Brazil Centro de Atenção Psicossocial Álcool e Drogas Butantã, São Paulo, SP, Brazil.; 4 Universidade Federal de São Paulo Departamento de Psiquiatria São Paulo SP Brazil Departamento de Psiquiatria, Universidade Federal de São Paulo, São Paulo, SP, Brazil.; 5 Centro Universitário Faculdade de Medicina do ABC Departamento de Neurociência Santo André SP Brazil Departamento de Neurociência, Centro Universitário Faculdade de Medicina do ABC, Santo André, SP, Brazil.; 6 Universidade de São Paulo Departamento de Psiquiatria, Faculdade de Medicina São Paulo SP Brazil Departamento de Psiquiatria, Faculdade de Medicina, Universidade de São Paulo (USP), São Paulo, SP, Brazil.; 7 Instituto Perdizes Hospital das Clínicas Faculdade de Medicina São Paulo SP Brazil Instituto Perdizes, Hospital das Clínicas, Faculdade de Medicina, USP, São Paulo, SP, Brazil.

**Keywords:** Brazil, mental health, services, LMIC, psycho-social

## Abstract

São Caetano do Sul, a city in southeastern Brazil, boasts exemplary social indicators and healthcare services, with a population of 162,763 and a density of 9,736.03 inhabitants/km². Allocating 25% of its budget to healthcare, the city's mental healthcare services adhere to the National Mental Health Policy. Structured services include a Center for Psychosocial Care (Centro de Atenção Psicossocial [CAPS])-II, CAPS-Alcohol and Drugs (CAPS-AD), outpatient teams, and teams in various locations. Initiatives since 2000 include inaugurating a CAPS-AD in 2006, a psychiatric emergency service in 2010, and a CAPS-II in 2016, relocating CAPS-AD in 2019, and establishing the Cuca Legal project in 2022. São Caetano do Sul has a Mental Health Risk Classification Protocol (Protocolo de Classificação de Risco à Saúde Mental) to aid clinical decision-making. Developing care lines for various groups, it offers programs like smoking prevention, school psychology, and obesity support. Collaborating with community centers, these facilities serve as teaching environments. The mental health care network focuses on five axes: Communication, Care, Prevention, Management, and Education, with specific proposed actions and competencies. Despite progress, challenges remain. Expanding access, reducing stigma, and implementing robust monitoring are crucial. São Caetano do Sul's experience offers valuable insights for similar urban settings in low- and middle-income countries (LMICs) developing mental health programs.

## Introduction

The municipality of São Caetano do Sul is located in the southeastern region of Brazil, specifically in the Metropolitan ABC region of São Paulo.^1^ It has a territorial area of 15,331 km², with an estimated population of 162,763 people and a population density of 9,736.03 inhabitants/km². São Caetano do Sul has the best social indicators in the entire country, and is considered an exemplary city in various aspects of the so-called Human Development Index (HDI) of the United Nations,^2^ ranking 1st on the list of Brazilian municipalities by HDI 0.862. The high quality of healthcare services is a distinguishing factor in the city of São Caetano. To maintain this benchmark status, the municipality allocates approximately 25% of its budget to healthcare,^3^ well above the minimum percentage required by the Federal Constitution, which is 15%.

Regarding mental healthcare services, the municipality is included in the Regional Plan of the Psychosocial Care Network (Plano Regional da Rede de Atenção Psicossocial [RAPS]) of the Metropolitan ABC region, Rede Regional de Atenção à Saúde (RRAS) 1,^4^ and has a structured network of mental healthcare services. Among the services offered and that make up the network are a CAPS-II, an extended CAPS-AD-II (there is an internal division of patients with CAPS and ambulatory profiles); two outpatient mental health teams (one type 2 and one type 3) for children at the Child and Adolescent Health Unit (Unidade de Saúde da Criança e do Adolescente [USCA]). In addition, there are mental health teams in the following locations: Family Health Support Centers (Núcleo de Apoio à Saúde da Família [NASF]),^5^ a service linked to primary care that identifies demands and provides support in the local territory, Integrated Centers for the Elderly (Centro Integrado de Saúde e Educação do Idoso [CISE]),^6^ Neonatal Screening and Sensory Stimulation Center (Centro de Triagem Neonatal e Estimulação Neurossensorial [CTNEN]), Comprehensive Women's Health Center (Centro de Atenção Integral à Saúde da Mulher [CAISM]), Center for Prevention and Care of Infectious Diseases (Centro de Prevenção e Assistência às Doenças Infecciosas [CEPADI]), municipal hospital complex, and municipal psychiatric emergency service. The actions of the mental healthcare teams are anchored in the National Mental Health Policy (Política Nacional da Saúde Mental), supported by Law 10.216/01,^7^ which seeks to consolidate an open and community-based mental healthcare model. This means ensuring the free circulation of people with mental disorders through services, territory, and the city, and offering care based on the resources that the community provides.

## Timeline

Prior to 2000, there was a mental health outpatient clinic with four psychiatrists offering individual psychological treatment without entry or discharge criteria, resulting in long waiting times. In February 2006, a CAPS-AD was inaugurated. In October 2010, a psychiatric emergency service was opened at Hospital Albert Sabin. In May 2016, a CAPS-II was inaugurated. In September 2019, the CAPS-AD was relocated to its own facility. In August 2020, the ambulatory mental health teams at USCA were qualified. In August 2022, the Cuca Legal project^8^ was established.

## Community involvement

The participation of the general public in the development of public policies for mental health is ensured through the implementation of municipal councils, such as the Municipal Council for Children and Adolescents (Conselho Municipal dos Direitos da Criança e do Adolescente [CMDCA]),^9,10^ Municipal Health Council (Conselho Municipal de Saúde [CMS]),^11^ Municipal Drug Policy Council (Conselho Municipal de Políticas sobre Drogas [COMAD]),^12^ Municipal Council for the Elderly (Conselho Municipal do Idoso [CMI]),^13^ and Municipal Council for People with Disabilities (Conselho Municipal da Pessoa com Deficiência [COMPED]).^14^ The city also holds municipal conferences on health and mental health, which include representation from various social segments, to assess the health situation and propose guidelines for the development of health policy at the municipal, state, and federal levels. Additionally, the services regularly hold assemblies with the users of the health facilities to deliberate on everyday decisions related to patient care.

## Mental Health Risk Classification Protocol

Mental health issues are chronic conditions that require continuous care within the healthcare system, at different levels of complexity and with the goal of providing comprehensive attention. Over time, these conditions can worsen and require specific and immediate interventions, calling for other points of attention beyond those already involved in continuous monitoring. Therefore, the municipality of São Caetano do Sul has established a Mental Health Risk Classification Protocol (Protocolo de Classificação de Risco à Saúde Mental), which serves as a tool to support clinical decision-making in regulating access to points of attention within the RAPS for acute cases. It is a clinical risk management process that aims to establish priorities for the care of mental health users who access the healthcare system and to define the most appropriate care resource for each case. As such, it aims to identify the most severe cases, allowing for a quicker and safer response according to the potential risk, health complications, or level of suffering. In addition to defining risk, the protocol also serves as a support for healthcare teams to assess the most suitable care resource for each case, seeking to improve referrals and patient follow-up within the healthcare network.

## Care dimensions

The care dimension is characterized by technical standardizations that explain information related to the organization of health actions offered by the system. It describes the patient's itinerary routines, including information regarding the actions and activities of promotion, prevention, treatment, and rehabilitation to be developed by a multidisciplinary team in each healthcare service. It enables communication between teams, services, and users in a health care network, focusing on action standardization and organizing a care continuum.

Regarding mental health care lines, the municipality of São Caetano do Sul is developing the care lines for self-inflicted violence, the LGBTQI+ population, and depression. The obesity care line has been in place in the municipality since 2020.

## Available programs

The network of mental health care services revolves around strategies adopted to achieve its objectives in preventing and treating the municipality's population with quality. Some programs offered by the services that make up the mental health network of São Caetano do Sul are highlighted in Table 1.

**Table 1:** Programs offered by the services that make up the mental health network of São Caetano do Sul

Program name	Description
Programa de Prevenção e Tratamento do Tabagismo^15-17^	Implementation of support groups and pharmacological intervention, with the help of a multidisciplinary team, to provide means to quit smoking. The service that was previously provided at the CAPS-AD (psychosocial care center) has been expanded, and now six UBSs conduct support groups, thus expanding access.^18-25^
Cuca Legal	School psychology – Institutional supervision work that offers support to the school technical staff in managing and preventing child and adolescent mental health in school units.
Programa Saúde nas Escolas^26^	A program that aims to contribute to the full development of public school students in basic education, through the coordination between primary care health professionals and education professionals.
Programa de Prevenção da Violência Infantil	Multidisciplinary care for children and adolescents who are victims of violence/sexual abuse residing in São Caetano do Sul, in the age range from zero to 17 years and 11 months and their families, aiming to prevent emotional, psychological, and psychiatric complications that affect the development of this specific population.
Juntas Somos Mais Fortes	Women in vulnerable situations – Constitution of an outpatient team in the perspective of expanding the offer of specialized mental health care to meet the demand for care for a public of women in situations of psychological suffering due to physical, sexual, and/or psychological violence.
INCLUARTE	Solidarity economy – Constitutes an alternative way to organize social relations according to principles that value the human being, work, justice, solidarity, and sustainability. This program is directed towards patients of CAPS-II and CAPS-AD in São Caetano do Sul.
Grupo Diário de Saúde	Groups that offer support and nutritional, sports, and psychological guidance, aiming to improve the quality of life of the city's population that is in an obesity situation.
Apoio e Orientação Familiar	Supporting, strengthening, and equipping families to fulfill their parental duties together with the State and society, in terms of protection and care provided to children and adolescents at each stage of development.

CAPS-AD = Centro de Atenção Psicossocial Álcool e Drogas; UBS = Unidade Básica de Saúde.

## Centers for health promotion

Health Promotion, according to the Ottawa Letter,^27^ encompasses five areas of action: the development and implementation of healthy public policies, the creation of healthy environments, community empowerment, the development of individual and collective skills, and the reorientation of health services.

Following this logic and understanding health in a broad sense, mental health teams collaborate with community centers for socializing, sports, leisure, and culture offered by the municipality of São Caetano do Sul (Table 2).

**Table 2:** Community centers for socializing, sports, leisure, and culture offered by the municipality of São Caetano do Sul

Facility name	Description
CISEs	Through joint actions in the areas of health, education, leisure, social issues, and citizenship, the CISEs are responsible for the care of the elderly population in the municipality that leads the country in terms of longevity, according to the Atlas of Human Development in Brazil (Atlas do Desenvolvimanto Humano no Brasil).^28^
Fundação das Artes	An institution of education and research in the arts that offers technical and free courses in the areas of visual arts, dance, music, and theater. It provides its students with experiences of artistic creation and dissemination aimed at collective practice, through cultural action in its headquarters and in various locations within and outside the city.
EMNOVA	The school, with its course offerings, fosters access to technology knowledge as a strategy for qualifying educational processes, professional development, solving family demands, and digital inclusion.^29^
Clubs	São Caetano do Sul has 14 municipalized clubs that offer sports activities in various modalities for the child, youth, and elderly population of the city.

CISE = Centro Integrado de Saúde e Educação do Idoso (Integrated Centers for the Elderly); EMNOVA = Escola Municipal de Novas Tecnologias (Municipal School of New Technologies).

## Health education

Mental health facilities also serve as teaching environments, and as such, they have a structured program and contracted services to allow residents to work alongside their supervisors throughout their training. These facilities receive medical interns, psychiatry residents, and interns from psychology, occupational therapy, and nursing programs.

## Mental health human resources

The administrative team of the Municipal Mental Health Program (Programa Municipal de Saúde Mental) includes two coordinators, three technical coordinators, three administrative coordinators, and three nursing technical supervisors. The technical team comprises 34 psychiatrists, 34 psychologists, four occupational therapists, one psycho-pedagogue, seven nurses, three nursing technicians, one nursing assistant, one nursing attendant, eight social workers, two pharmacists, two pharmacy assistants, four workshop facilitators, six janitors, and four security guards.

## Axes of interventions

The actions proposed to achieve the goals of the mental health care network in the municipality are divided into five axes: Communication, Care, Prevention, Management and Guidelines and Permanent Education (Table 3).

**Table 3:** Mental health axes in São Caetano do Sul

Proposed actions		Competence
Communication axis		
	Conduct awareness campaigns for the population about prevention and symptoms of mental health problems through social media and printed materials.	Communications Section of the Health Department (Seção de Comunicação da Secretaria de Saúde) / Mental Health Section of the Health Department (Seção de Saúde Mental da Secretaria de Saúde)
	Establish a frequency for publishing posts on city hall social media with content aimed at providing clarification on prevention and symptoms.	Communications Section of the Health Department (Seção de Comunicação da Secretaria de Saúde) / Mental Health Section of the Health Department (Seção de Saúde Mental da Secretaria de Saúde)
	Produce graphic materials for health units such as posters and leaflets with content focused on prevention and symptoms.	Communications Section of the Health Department (Seção de Comunicação da Secretaria de Saúde) / Mental Health Section of the Health Department (Seção de Saúde Mental da Secretaria de Saúde)
	Semana Municipal de Saúde Mental	Communications Section of the Health Department (Seção de Comunicação da Secretaria de Saúde) / Mental Health Section of the Health Department (Seção de Saúde Mental da Secretaria de Saúde)
	Keep information updated on city hall portals.	Communications Section of the Health Department (Seção de Comunicação da Secretaria de Saúde) / Mental Health Section of the Health Department (Seção de Saúde Mental da Secretaria de Saúde)
Assistance axis		
	Registration of 100% of CAPS users in the strategic health program of the primary care family health (AB).	Specialized services/AB
	Increased active search for patients.	Specialized services/AB
	Expansion of the multidisciplinary care offer.	Health Department (Secretaria de Saúde), especially the Mental Health Section (Seção de Saúde Mental)
	Strengthening the socio-assistance network aimed at socio-family monitoring and inclusion of children, adolescents, and young people who use alcohol and other drugs in social reintegration programs.	Department of Assistance and Social Integration (Secretaria de Assistência e Integração Social)
	Encouraging the creation and participation in vocational training courses, leisure, cultural, and sports activities.	Department of Health, Education, Assistance and Social Integration, and Culture (Secretaria de Saúde, Educação, Assistência e Integração Social e Cultura)
	Strengthening the solidarity economy in the social inclusion and work of alcohol and other drug users.	Specialized services
Preventive axis		
	Provide continuous drug prevention programs for children and adolescents in the education system.	Department of Health, Education, Safety (Secretaria de Saúde, Educação, Segurança), and Conselho Municipal de Drogas (City Drug Counsel)
	Identify and discuss cases of children and/or adolescents with learning, emotional, behavioral problems, and their family dynamics in a network.	Connected territory
	Strengthen the discussion of issues related to human development, social vulnerability, and topics related to drug use as a transversal and interdisciplinary theme within the school curriculum.	Department of Health and Education (Secretaria de Saúde e Educação)
Management and guidelines axis		
	Annually conduct educational contests on the theme of alcohol and other drugs in the education system.	Department of Health, Education (Secretaria de Saúde, Educação), and Conselho Municipal de Drogas (City Drug Counsel)
	Develop an intersectoral action plan to incorporate leisure, cultural, and sports activities in areas of greater vulnerability, aiming to prevent the use of psychoactive substances and mental health issues.	Health Department (Secretaria de Saúde), especially the Mental Health Section (Seção de Saúde Mental)
	Strengthen the Municipal Mental Health Program (Programa Municipal de Saúde Mental).	Health Department (Secretaria de Saúde)
	Annually update and publicize the list of services provided by the municipality's RAPS.	Mental Health Section of the Health Department (Seção de Saúde Mental da Secretaria de Saúde)
Continued education axis		
	Ensure a space for sharing and monthly meetings to exchange experiences among professionals in the intersectoral service network.	Mental Health Section of the Health Department (Seção de Saúde Mental da Secretaria de Saúde)
	Ensure that the decentralization of services occurs according to the demand's needs.	Mental Health Section of the Health Department (Seção de Saúde Mental da Secretaria de Saúde)
	Create flows and procedures for the joint action of health, social assistance, and education services and workers, to be validated and agreed upon by workers, managers, and service users.	Mental Health Section of the Health Department (Seção de Saúde Mental da Secretaria de Saúde)
	Encourage the participation of representatives from civil society through broad dissemination of the Councils and educational campaigns on popular participation.	Mental Health Section of the Health Department (Seção de Saúde Mental da Secretaria de Saúde)
	Ensure the training of councilors (representatives of government and civil society) through participation in congresses, courses, lectures, forums, etc.	Mental Health Section of the Health Department (Seção de Saúde Mental da Secretaria de Saúde)
	Ensure that any legislation project related to the theme is submitted for consideration to the Legislative Chamber.	Mental Health Section of the Health Department (Seção de Saúde Mental da Secretaria de Saúde)
	Ensure the ongoing training of specialized health teams and primary care.	Health Department (Secretaria de Saúde), especially the Mental Health Section (Seção de Saúde Mental)
	Mobilize and ensure the participation of staff in courses, seminars, lectures, etc., related to the subject.	Health Department (Secretaria de Saúde), especially the Mental Health Section (Seção de Saúde Mental)
	Promote educational activities with the staff.	Health Department (Secretaria de Saúde), especially the Mental Health Section (Seção de Saúde Mental)

AB = Atenção Básica (primary care); CAPS = Centro de Atenção Psicossocial; RAPS = Plano Regional da Rede de Atenção Psicossocial.

## Overview of consultations

During the period from January 1 to December 8, 2023, the CAPS-II "Dr. Ruy Penteado" provided a total of 35,670 consultations across various specialties within the technical team. These specialties encompassed psychiatry, psychology, occupational therapy, social work, nursing, and workshop facilitators, with specific consultations as follows: psychiatry (7,980 consultations), psychology (13,026 consultations), occupational therapy (2,588 consultations), social work (2,878 consultations), nursing (6,653 consultations), and workshop facilitators (2,545 consultations).

Similarly, during the same period, the CAPS-AD "Zoraide Maria Rampasso" conducted a total of 14,927 consultations within its specialized areas. The breakdown of consultations within CAPS-AD included psychiatry (1,858 consultations), psychology (3,223 consultations), occupational therapy (1,189 consultations), social work (2,057 consultations), nursing (3,737 consultations), pharmacy (1,037 consultations), and workshop facilitators (1,826 consultations).

Furthermore, the mental health team at the USCA provided a total of 18,575 consultations during the same period. These consultations encompassed child psychiatry (1,725 consultations), psychology (13,183 consultations), social work (1,789 consultations), and psycho-pedagogy (1,878 consultations).

In aggregate, the three facilities collectively administered 69,172 consultations. Notably, this figure does not account for consultations conducted by mental health teams within hospital psychology and psychiatry departments, CEPADI, CTNEN, CISE, and CAISM. Additionally, it excludes activities carried out by school psychology teams.

## Discussion

The comprehensive overview of São Caetano do Sul's mental healthcare system provided in this manuscript underscores the municipality's commitment to ensuring the well-being of its residents. Through strategic planning, community involvement, and a multidisciplinary approach, São Caetano do Sul has developed a robust network of mental health services that prioritize accessibility, quality of care, and continuous improvement.

When comparing São Caetano do Sul's mental healthcare system with those of other municipalities or regions, several key strengths emerge. One notable strength is the municipality's high level of integration within the broader healthcare infrastructure. Unlike some regions where mental health services may operate in isolation or with limited coordination, São Caetano do Sul has successfully aligned its mental health services with national and regional healthcare policies. This integration allows for seamless collaboration between different healthcare facilities and professionals, facilitating holistic care for individuals with mental health conditions.

Furthermore, São Caetano do Sul's emphasis on community involvement sets it apart from many other municipalities. By establishing municipal councils and regularly engaging service users in decision-making processes, São Caetano do Sul demonstrates a commitment to participatory governance and stakeholder engagement. In contrast, some regions may lack robust mechanisms for community input, leading to a disconnect between mental health policies and the needs of the population.

Another area of comparative analysis lies in the implementation of innovative practices and protocols. São Caetano do Sul's adoption of the Mental Health Risk Classification Protocol represents a significant advancement in the management of acute mental health cases within the healthcare system. While similar protocols may exist in other regions, São Caetano do Sul's proactive approach to risk assessment and intervention sets a benchmark for evidence-based practices in mental healthcare delivery.

Additionally, São Caetano do Sul's focus on health promotion and disease prevention aligns with global recommendations for comprehensive mental health care. Compared to regions that primarily emphasize reactive treatment approaches, São Caetano do Sul's investment in preventive measures and health education reflects a forward-thinking strategy aimed at addressing the root causes of mental illness and promoting overall well-being.

In conclusion, São Caetano do Sul's mental healthcare system stands out for its integration, community involvement, innovative practices, and emphasis on prevention. While there may be variations in mental healthcare delivery across different regions, São Caetano do Sul serves as a model for effective and inclusive mental health service provision. Moving forward, continued efforts to evaluate, adapt, and share best practices will be essential for advancing mental health agendas globally and ensuring that all individuals have access to the care and support they need.

## Future directions

While São Caetano do Sul's mental health network has made significant progress in recent years, there are still some limitations that need to be addressed to ensure that mental health services are accessible and effective for all residents.

One limitation is the availability of mental health services outside the central region of the city. While the mental health network comprises a CAPS-II, an extended CAPS-AD-II, outpatient mental health teams, and mental health teams in various locations, it is important to ensure that all residents have access to mental health services, regardless of their location in the city. One potential solution could be to increase the number of mental health teams in remote areas and to strengthen partnerships with community centers and other local organizations to improve access to mental health services.

Another limitation is the need to address the stigma surrounding mental health issues in the city. Despite the efforts of the mental health network to raise awareness and promote mental health initiatives, stigma and discrimination remain significant barriers to mental health care in São Caetano do Sul. Future initiatives should focus on promoting education and awareness campaigns to reduce stigma and discrimination related to mental health issues.

Finally, the mental health network of São Caetano do Sul could benefit from a more robust system for monitoring and evaluating the quality and effectiveness of mental health services. While the municipality has established a Mental Health Risk Classification Protocol to support clinical decision-making for acute cases, there is a need for a more comprehensive monitoring and evaluation system to ensure that mental health services are meeting the needs of residents and achieving the desired outcomes. Such a system could help identify areas for improvement and ensure that mental health services are continually evolving to meet the changing needs of the community.

## Conclusion

While São Caetano do Sul's mental health network has made impressive strides in recent years, there are still some limitations that need to be addressed. By expanding access to mental health services, reducing stigma and discrimination, and implementing a robust system for monitoring and evaluation, São Caetano do Sul can continue to improve its mental health network and provide high-quality, accessible mental health care to all residents.

## Data Availability

Not applicable.
